# Hands-Off Time for Endotracheal Intubation during CPR Is Not Altered by the Use of the C-MAC Video-Laryngoscope Compared to Conventional Direct Laryngoscopy. A Randomized Crossover Manikin Study

**DOI:** 10.1371/journal.pone.0155997

**Published:** 2016-05-19

**Authors:** Philipp Schuerner, Bastian Grande, Tobias Piegeler, Martin Schlaepfer, Leif Saager, Matthew T. Hutcherson, Donat R. Spahn, Kurt Ruetzler

**Affiliations:** 1 Institute of Anesthesiology, University and University Hospital Zurich, Zurich, Switzerland; 2 Departments of Outcomes Research and General Anesthesiology, Cleveland Clinic, Cleveland, Ohio, United States of America; Azienda Ospedaliero-Universitaria Careggi, ITALY

## Abstract

**Introduction:**

Sufficient ventilation and oxygenation through proper airway management is essential in patients undergoing cardio-pulmonary resuscitation (CPR). Although widely discussed, securing the airway using an endotracheal tube is considered the standard of care. Endotracheal intubation may be challenging and causes prolonged interruption of chest compressions. Videolaryngoscopes have been introduced to better visualize the vocal cords and accelerate intubation, which makes endotracheal intubation much safer and may contribute to intubation success. Therefore, we aimed to compare hands-off time and intubation success of direct laryngoscopy with videolaryngoscopy (C-MAC, Karl Storz, Tuttlingen, Germany) in a randomized, cross-over manikin study.

**Methods:**

Twenty-six anesthesia residents and twelve anesthesia consultants of the University Hospital Zurich were recruited through a voluntary enrolment. All participants performed endotracheal intubation using direct laryngoscopy and C-MAC in a random order during ongoing chest compressions. Participants were strictly advised to stop chest compression only if necessary.

**Results:**

The median hands-off time was 1.9 seconds in direct laryngoscopy, compared to 3 seconds in the C-MAC group. In direct laryngoscopy 39 intubation attempts were recorded, resulting in an overall first intubation attempt success rate of 97%, compared to 38 intubation attempts and 100% overall first intubation attempt success rate in the C-MAC group.

**Conclusion:**

As a conclusion, the results of our manikin-study demonstrate that video laryngoscopes might not be beneficial compared to conventional, direct laryngoscopy in easily accessible airways under CPR conditions and in experienced hands. The benefits of video laryngoscopes are of course more distinct in overcoming difficult airways, as it converts a potential “blind intubation” into an intubation under visual control.

## Introduction

Airway Management is essential in patients suffering from cardiac arrest who are undergoing cardio-pulmonary resuscitation (CPR)[1, 2]. According to current CPR guidelines, rescuers can choose from the wide variety of airway devices and ventilation techniques available in the current CPR guidelines, airway management during CPR is an on-going area of debate. However, endotracheal intubation remains the standard approach for securing and maintaining a patent airway during CPR,[1, 3, 4] which requires highly skilled and experienced personnel, along with regular training and practice. In addition, supplementary tools[5–9] are sometimes used to minimize the time of interruption of the chest compressions (termed “hands-off time”) and to avoid unrecognized esophageal intubation, which can result in subsequent catastrophic clinical consequences.[1, 10]

Video laryngoscopes have been introduced to allow monitoring and to assist tracheal intubation when visualization of the glottis is difficult.[11] Recent studies confirmed that video laryngoscopes might offer better views of the glottis when compared with direct laryngoscopy and therefore may serve as a valid alternative option for the management of the expected and unexpected difficult airway.[12–16] Thus, video laryngoscopes have gained an important role in the management of patients with (unanticipated) difficult or failed endotracheal intubation.

The impact of uninterrupted, high-quality chest compressions during CPR for patient outcomes is undisputed, as they are essential for maintaining vital organ perfusion.[17] Minimizing hands-off time by the introduction of special training drills resulted in an up to 3-fold increase in survival of out-of-hospital cardiopulmonary arrest.[18] Consequently, current CPR guidelines highlight the fact that interruptions of chest compressions during CPR should be as short as possible.[1, 3] Skilled clinicians should be able to fully secure the airway without interrupting chest compressions or within a brief pause of less than 5 seconds.[1]

Several studies suggest that visualization of the vocal cords and subsequent endotracheal intubation might be faster and more successful during the initial intubation attempt if using a video laryngoscope, compared to conventional direct laryngoscopy.[19] We thus used a manikin model to determine whether endotracheal intubation during CPR would be faster and more successful using a video laryngoscope compared to direct laryngoscopy.

## Materials and Methods

With approval of the local Ethics Committee (Kantonale Ethikkommission Zurich—application number 16/13, chair Prof. Peter Meier-Abt) and written informed consent, 26 anesthesia residents and 12 anesthesia attending physicians of the University Hospital Zurich were recruited between June and August 2014. All participants had performed more than 150 tracheal intubations, a number which has been shown to be sufficient to reach an overall success rate of over 90%.[20] Furthermore, none of the participants had any previous experience with video laryngoscopy. This may be somewhat surprising, but fiberoptic bronchoscopes are more widely used in our institution than video laryngoscopes. The number of study participants was a result of the voluntary enrollment, thus a sample size calculation and power analysis were not performed.

### Study protocol

All participants attended a standardized day-long, hands-on seminar covering relevant aspects of CPR. At the end of the seminar, the C-MAC video laryngoscope was introduced to the participants, explained, and demonstrated using an advanced patient simulator (Resusci Anne Advanced Simulator, Laerdal Medical, Stavanger, Norway). This airway management trainer allows simulation of a normal airway and is widely used as an effective learning tool. The manikin was placed dorsal on a standard operating table. Participants were instructed to intubate the manikin during on-going chest compressions and were randomly assigned to one of two groups using the following airway tools:

1. Direct laryngoscopy (Macintosh blade size 3), endotracheal intubation with a 7.5 mm I.D. tube (Mallinckrodt, Athlone, Ireland), reinforced with a stylet.
2. C-MAC video laryngoscope with D-blade size 3 (Karl Storz, Tuttlingen, Germany), endotracheal intubation with a 7.5 mm I.D. tube (Mallinckrodt, Athlone, Ireland), reinforced with a stylet.

Randomization was based on two identical papers labeled as either C-MAC or laryngoscopy, which were placed face down on the table and selected by the participants. A participant not involved in this study performed chest compressions in accordance to current CPR guidelines. After 30 seconds of chest compressions, the participants were asked to perform the endotracheal intubation.

The participants were strictly advised to perform endotracheal intubation during on-going chest compressions to avoid any airway management-associated hands-off time. If necessary, participants were allowed to ask for a pause of the on-going chest compressions, but were asked to allow only minimal hands-off time. Repositioning of the airway device was allowed only if the participants recognized a poor-positioning themselves. Airway management was considered complete when the study device was inserted and the manikin could be ventilated successfully. If more than three attempts were required or in the instance of unrecognized esophageal intubation, airway management was stopped and defined as failure. After finishing the first intubation scenario, participants were asked to perform airway management with the alternate intubation technique (C-MAC video laryngoscope or direct laryngoscopy).

The manikin’s airway, the tracheal tubes, and intubation stylets were lubricated thoroughly with a lubricant recommended by the manufacturer (Laerdal Airway Lubricant, Laerdal Medical, Stavanger, Norway). To avoid any teaching bias, all particpants underwent individual evaluation and their peers were not allowed to watch.

### Measurements

The primary outcome parameter was hands-off time, defined as the cumulative duration of CPR discontinuation during airway insertion.[21] A discontinuity of chest compressions during airway management exceeding one second was considered to be the beginning of a hands-off period. The manikin’s computer automatically recorded cumulative hands-off time during airway management episode(s), with no requirement that the episodes be contiguous.

Successful intubation, number of intubation attempts, and subsequent first intubation attempt success rate were determined by an investigator and provided additional secondary outcome parameters for analysis. After completion of the intubation scenario, participants were asked to rate the ease of intubation using the C-MAC or the direct laryngoscopy (1 very easy, 2 easy, 3 moderate, 4 somewhat difficult, 5 impossible).

### Statistical analysis

The Wilcoxon rank-sum test was used to compare hands-off time, intubation attempts, and ratings. Hands off time and ratings are presented as mean time ± standard deviation (SD). Intubation attempts are presented as absolute values. A p-value below 0.05 was considered to be statistically significant. All analyses were performed with the SigmaPlot 11.0 (Systat Software Inc., Erkrath, Germany).

## Results

**Fig 1:**
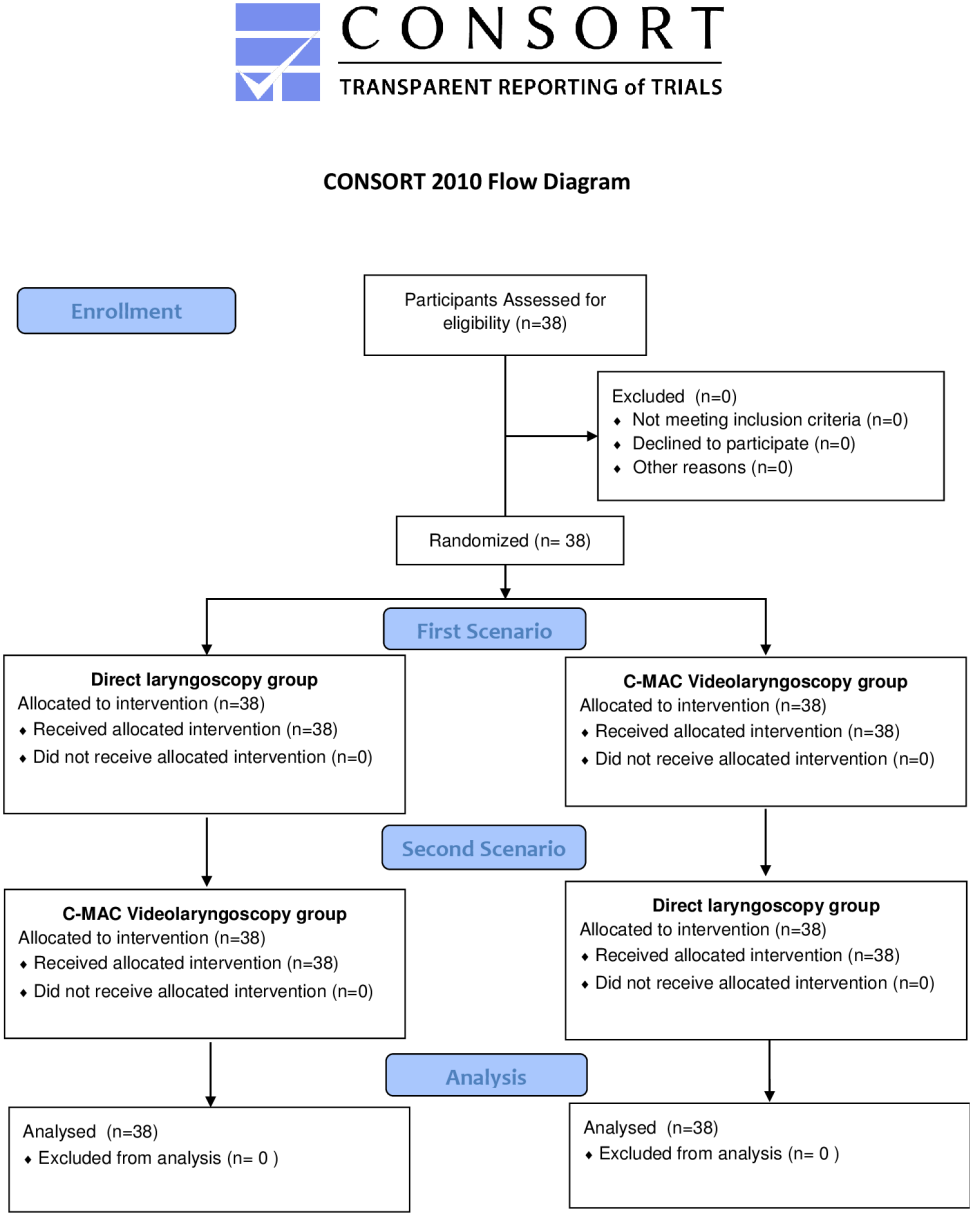
CONSORT Diagram.

26 residents with at least one year of clinical experience and 12 attending anesthesiologists (a total of 14 women and 24 men, age 29 ± 4 years) participated in this study. The results of all 36 intubations using C-MAC and laryngoscope were available for statistical analysis (Fig 1).

The mean hands-off time using direct laryngoscopy was 1.9 ± 2.1 seconds, whereas mean hands-off time for C-MAC was 3.0 ± 2.7 seconds; p = 0.048 (Table 1).

**Table 1:** Hands-off time, intubation attempts, first intubation attempt success rate and rating.

	hands-off time [s]	intubation attempts [n]	First intubation attempt success rate [%]	rating
laryngoscope	1.9 ± 2.1	39	97%	1.9 ± 0.7
C-MAC	3.0 ± 2.7	38	100%	1.5 ± 0.6
p-value	**0.048**	0.33		**0.009**

Thirty-nine intubations attempts were necessary in the direct laryngoscopy and 38 in the C-MAC group, resulting in a first intubation success rate of 97% in direct laryngoscopy group (37 out of 38 intubations) compared to 100% in C-MAC group (38 out of 38 intubations) (Table 1).

The participants rated the direct laryngoscopy guided intubation significantly more difficult than the C-MAC guided intubation (1.9 ± 0.7 versus 1.5 ± 0.6; p = 0.009) (Table 1).

## Discussion

The aim of this study was to compare hands-off time associated with two different airway management tools during on-going chest compressions in a manikin setting. The main finding of our study is that hands-off time (1.9 versus 3.0 seconds) and success rate (97 versus 100%) were comparable between direct laryngoscopy and C-MAC video laryngoscopy guided endotracheal intubation. Although this difference was statistically significant, the mean difference of 1 second is likely to be clinically irrelevant.

The value of uninterrupted high-quality chest compressions is undisputed.[17] Interruptions in chest compressions are known to decrease coronary perfusion pressure, reduce return of spontaneous circulation (ROSC), and are associated with decreased defibrillation success and poor outcome.[22, 23] According to CPR guidelines, chest compressions should be discontinued only in order to pass the endotracheal tube through the vocal cords and only if considered necessary by the person performing intubation.[1, 3]

Several studies investigated the potential impact of airway management and the associated hands-off time.[2, 21, 24–26] One of the few clinical studies available, published by Wang et al[21], observed a median time to intubation of 46 seconds and substantial pauses in chest compressions of 109 seconds. Overall, the facilitation of endotracheal intubation contributed to nearly 23% of all CPR interruptions recorded.[21] The majority of the studies investigating hands-off time were performed in manikins instead of real patients. However, the data consistently demonstrated that airway-associated hands-off time is clearly associated with the type of airway device used and the experience of the provider: the more experienced the provider was with the respective airway device, the shorter the resulting hands-off time.

In our study setting, the providers were highly experienced in direct laryngoscopy using a Macintosh blade, as this represents our first line intubation device. However, they were not familiar with the C-MAC video laryngoscope, although this lack of experience might be negligible due to the fact that the blade of the C-MAC video laryngoscope is identical to the conventional Macintosh blade. As a consequence, the overall medium hands-off time was nearly identical in both study groups.

These results confirm prior findings by Park et al., where the authors retrospectively analyzed 71 cardiac arrest patients undergoing CPR in an emergency department and reported no significant interruptions of chest compressions using the Glide Scope video laryngoscope.[27] The authors also reported a first attempt intubation success rate of 93%, which is in accordance with the findings of our study. The first attempt intubation success rate in our study was 97% (direct laryngoscopy), and 100% (C-MAC), respectively. However, this may also have been affected by the high level of experience of the providers included in our study.

Another study investigated airway management-associated hands-off time in adult patients undergoing in-hospital CPR.[22] The authors reported, that endotracheal intubation was associated with 15 seconds of interrupted hands-off time, compared to 8 seconds placing a laryngeal mask.[22]

Interestingly, a recent study also demonstrated that at least in the infant CPR manikin setting, the use of a video laryngoscope might not be advantageous compared to standard (direct) laryngoscopy during on-going chest compressions in terms of time to intubation and failure to intubate within 30, 45 and 60 seconds.[28] Although this study was performed in an infant CPR manikin setting, it may still confirm the findings of our study.

Several publications investigating a wide range of airway devices demonstrated that as soon as the airway device was successfully placed, overall hands-off time was reduced, mostly due to switching from 30:2 to ongoing chest compressions.[26, 29]

Our study has several limitations. This is a manikin study, which may not adequately mimic the human airway under real CPR conditions. However, due to ethical considerations, this cross over study might be not feasible in a real CPR setting. However, the advantage of using manikins is that we could use a cross-over design and thus provide standardized airway conditions for each participant. Nevertheless, findings of manikin studies can be less reliable in real life and need to be confirmed in “real patients”.

In conclusion, the results of our study demonstrate that the C-MAC video laryngoscope was not superior compared to conventional, direct laryngoscopy in securing airways under CPR conditions. The results of this study are limited by the fact, that this study was performed in manikins and that intubation well-trained providers performed endotracheal intubation. However, although this was not specifically investigated in this study, the use of video laryngoscopy, such as the C-MAC, is probably not necessary in easy-to-handle airways, but may be more beneficial in overcoming difficult airways, as it converts a potential “blind intubation” into an intubation under visual control.

## Supporting Information

S1 CONSORT Checklist (DOC)
